# A Few Diseases of Horses

**Published:** 1887-08

**Authors:** J. Hall

**Affiliations:** Toronto; Bureau Clinical Medicine, I. H. A.


### A FEW DISEASES OF HORSES, WITH SOME REMARKS ON THE PLEURO-PNEUMONIA OF CATTLE.

J. HALL, SR., M. D., TORONTO.

(Bureau Clinical Medicine, I. H. A.)

My first horse was a black Canadian pony, seeming completely sound, and answering my purpose well. But after awhile had a severe attack of pain in the bowels—so severe that he laid down on his belly or side, kicking about so desperately that it was no easy matter to approach and examine him—the bowels seemed confined and urine high colored, and being afraid of inflammation, which I could not make out, consented to send for a veterinary surgeon to give me his diagnosis of the case. He soon came, and almost as quickly said the disease was colic, on which I proposed to take the treatment into my own hands, to which, however, he strongly dissented, saying I would soon kill the horse, etc. I therefore reluctantly consented that he should manage the treatment, the first thing done being an injection per annum to bring away the offending matter, and that not sufficing, another and another, followed by cathartic medicines to empty the bowels and so give relief from pain; but, notwithstanding all this, the poor horse remained on his side or belly some two days, groaning and moaning most piteously with his sufferings. At this stage I dismissed the veterinary surgeon, determining to assume the responsibility myself, for to all appearances the animal was doomed, notwithstanding this most heroic treatment.

To antidote these abuses, and as a means of overcoming his malady, I put him on Nux vom.$^{2c}$—some thirty pills—every hour until relief could be obtained, which occurred about the fourth dose, when the horse was so relieved that he got up and began shortly afterward to eat, and two days after this was at his work. Of course, the veterinary surgeon never forgave me, but alluded to our treatment so severely in his lectures that one morning, a long time afterward, two men called on me, and, with a smile on their faces, said they had come to learn if there was really any truth in Homœopathy. I at once replied that they must think me an impostor to spend all my time in the study and practice of this art when I had no confidence in it. They then frankly told me that their professor took such pains in almost all his lectures to malign and kill Homœopathy, that they thought there must be something in it, and had come to me to ascertain the facts. I was at once interested and took occasion to show what true Homœopathy was, and I gave them every information in my power on this subject, and I had the satisfaction of making two homœopaths of these students, who are now in active practice as homœopathic veterinary surgeons, and can only hope that they are genuine, knowing what our true science is.

My second case was also a black horse of a very fine breed, but sold to me at a low price after his head and neck had been sadly disfigured and permanently injured by blisters for the epizootic, which were thought by some to be the treatment for that disease. This animal so bought and disfigured seemed always in trouble, as if something annoyed him—holding back his ears when traveling, or biting his groom or any person who came near, and it was some time before his real malady manifested itself, being overlooked by his disposition. But at length, noticing that the hair of his mane and tail was very thin and daily becoming more so, I found on examination that he suffered severely from a pruritus, being constantly rubbing both tail and mane to get ease; but having no very definite symptoms to go upon gave him Sulph.$^{2c}$, three or four doses, and after ten days 20M and then 40M, waiting their action, which was only partial. Being nonplussed, I had thought the case difficult and, perhaps, incurable; but observing that the horse was always very chilly, not bearing the cold at all, I gave him Silicea$^{2c}$, several doses, and with the best effects; and after two weeks Silicea$^{20m}$, two or three doses, and still after that some weeks, Sil.$^{40m}$, one dose, and, perhaps once, Silicea$^{cm}$, taking about six or eight weeks in all, after which the animal was cured, both tail and mane growing nicely and very full, while the horse's skin, which was always rough, had become smooth, and chilliness had disappeared, holding both head and ears when driving as if perfectly satisfied and happy. My stable-boy thinks that good grooming had much to do with it, but he had that before and behind too, and was, moreover, subject to relapses, when another dose of medicine was given him. I must confess that, although the brute had no faith in me or my little pills, I had it in unbounded measure, and gave them all the credit.

Third case was the horse of my partner, Dr. J. W. Hunter Emory, a brown gelding of excellent constitution. One day when Dr. E. had left the horse in my yard tied to a weight, the animal, liking always to have his own way, and perhaps thinking the groom rather tardy in giving him his dinner, took it into his head to walk into the street, drawing the weight and sleigh after him, which, being somewhat cumbersome, he reared up highly, breaking a shaft thereby, and running some eight or ten inches of the broken part into his rectum, the pain of which being very acute, the animal fairly screamed, and was quietly led into the stable. Here was a difficulty indeed, and the diagnosis not easy, and a veterinary surgeon was suggested, which I strongly opposed, as these men would spend all their time and knowledge in finding out what was the matter and the extent of the injury. The first day Arnica$^{2c}$ was given every two hours, then, Calendula$^{2c}$ every four hours, after which what should come next was a question. The horse had not dunged since the accident, and could not, but stood trembling in his stall, making unsuccessful efforts, but dare not push much, when Dr. E., who, being a good surgeon, used a fountain syringe to the part and succeeded in removing a large amount of fæcal matter, while the pus flowed most abundantly, and so very fetid that one could hardly remain in the stable. On the whole, on account of the very fetid pus, Silicea$^{2c}$ was given for two days every four hours, with very great mitigation of the odor, and the horse began to eat and to pass his dung, and after five or six days Silicea$^{20m}$ was next given, in three doses, and after this, say in three weeks, the horse was at his work, the groom reporting him well. There is, however, a very little pus still excreted at times, and the part affected looks like a fistula, which Dr. E. thought was the sole trouble at first; a few doses of Sulphur or Thuja will now be tried and their effects recorded, as a cure is certain.

Fourth case was a horse of my partner's father, which had been for some twelve months very much troubled with lumbrici, for which he had received, according to the old-school treatment, repeated doses of physic, or cathartic medicine, to take the vermin away, which, indeed, it did, but they as certainly came again, when the dose had to be repeated, until the horse was reduced to a mere skeleton by this twelve months' treatment, looking much as if he would die. It was at this stage that the animal was seen by Dr. Emory, who induced his parent to try true Homœopathy on the animal, to which consent being given, he was put upon Nux vom.$^{2c}$ morning and night for a week, and after this Cina$^{2c}$ in the same way; since which the animal has perfectly recovered his normal condition, being no more troubled with lumbrici, nor asking any questions as to his cure.

At the risk of a slight digression, I may here remark that it is a sad pity some true and good men of our school do not take hold of that terrible plague of cattle called pleuro-pneumonia, whose victims are so numerous and their loss equally heavy on holders, for I firmly believe that should such tackle it, the deaths would be very rare indeed. There is great talk of stamping it out, which the old school will never do, but such a procedure on our part would effectually do it. The epizootic or malignant influenza is also perfectly and quickly manageable, as my own experience abundantly proves.

